# Endemicity and evolutionary value: a study of Chilean endemic vascular plant genera

**DOI:** 10.1002/ece3.960

**Published:** 2014-02-19

**Authors:** Rosa A Scherson, Abraham A Albornoz, Andrés S Moreira-Muñoz, Rafael Urbina-Casanova

**Affiliations:** 1 Departmento de Silvicultura y Conservación de la Naturaleza, Facultad de Ciencias Forestales y Conservación de la Naturaleza, Universidad de ChileSanta Rosa, 11315, Santiago, Chile; 2 Center for Advanced Studies in Ecology and Biodiversity (CASEB), Pontificia Universidad Católica de ChileCasilla 114-D, Santiago, Chile; 3 Instituto de Geografía, Pontificia Universidad Católica de ChileAv. Vicuña Mackenna, 4860, Santiago, Chile

**Keywords:** Community structure, endemicity, phylogenetic diversity, vascular plants

## Abstract

This study uses phylogeny-based measures of evolutionary potential (phylogenetic diversity and community structure) to evaluate the evolutionary value of vascular plant genera endemic to Chile. Endemicity is regarded as a very important consideration for conservation purposes. Taxa that are endemic to a single country are valuable conservation targets, as their protection depends upon a single government policy. This is especially relevant in developing countries in which conservation is not always a high resource allocation priority. Phylogeny-based measures of evolutionary potential such as phylogenetic diversity (PD) have been regarded as meaningful measures of the “value” of taxa and ecosystems, as they are able to account for the attributes that could allow taxa to recover from environmental changes. Chile is an area of remarkable endemism, harboring a flora that shows the highest number of endemic genera in South America. We studied PD and community structure of this flora using a previously available supertree at the genus level, to which we added DNA sequences of 53 genera endemic to Chile. Using discrepancy values and a null model approach, we decoupled PD from taxon richness, in order to compare their geographic distribution over a one-degree grid. An interesting pattern was observed in which areas to the southwest appear to harbor more PD than expected by their generic richness than those areas to the north of the country. In addition, some southern areas showed more PD than expected by chance, as calculated with the null model approach. Geological history as documented by the study of ancient floras as well as glacial refuges in the coastal range of southern Chile during the quaternary seem to be consistent with the observed pattern, highlighting the importance of this area for conservation purposes.

## Introduction

The maintenance of the evolutionary and ecological processes that guarantee the sustainability of biological diversity has been identified as the main goal of any action directed to the protection of natural environments (Purvis et al. 2005). In the face of limiting resources, the need to prioritize efforts in order to guarantee efficient conservation actions is of great importance. The identification of those taxa and areas that should be conservation priorities has been long known as the “agony of choice” (Vane-Wright et al. 1991) or the “conservation resource allocation problem” (Wilson et al. 2006). One of the characteristics of current times that complicates the classification and management of taxa and ecosystems is the uncertainty as to the conditions, especially climatic, that taxa will face in the mid and long term. Global change processes have sped up with respect to historic registries, and several possible scenarios of climatic change are considered that taxa and ecosystems will have to face in the future (Midgley et al. 2002). Several measures have been developed to quantitatively estimate the “value” of taxa and ecosystems. Currently, traditional richness data coupled with information on evolutionary patterns have been suggested as a meaningful measure of biodiversity aimed at preserving the maximum amount of character diversity, in the face of an uncertain future (Vane-Wright et al. 1991; Sechrest et al. 2002; Fisher and Owens 2004; Faith and Baker 2006; Forest et al. 2007; Pio et al. 2011). Given limited resources, conservation efforts should arguably be directed toward taxa and/or areas that represent the greatest amount of unique evolutionary history (Collen et al. 2011).

The most commonly used evolutionary measure given a phylogeny with branch lengths is phylogenetic diversity (PD; Faith 1992). Basically, PD measures the accumulation of attributes or adaptations in a taxon or a group of taxa and provides a quantitative idea of how much evolutionary history would be lost if those taxa were not preserved (Faith 1992; Purvis 2000). PD is calculated by summing the branches that connect the target taxa to either the root of the subtree, PD_NODE_ (Faith 1992) or the root of the whole phylogeny, PD_ROOT_ (Rodrigues and Gaston 2002). The PD index is particularly significant as it considers the accumulated evolution of a set of taxa and hence its evolutionary potential (Forest et al. 2007; Potter 2008), but see Winter et al. (2013) for discussion on this topic. It has also been shown that PD of a community is positively correlated with ecosystems primary productivity (Cadotte et al. 2009). Whether or not species richness is a good surrogate for PD has been argued, and it is currently recognized that this is not always the case, given the multiple processes that affect speciation, extinction, and radiation in an area (Rodrigues and Gaston 2002; Tôrres and Diniz-filho 2004; Forest et al. 2007; McGoogan et al. 2007; Pio et al. 2011; Scherson et al. 2012). In addition to PD, phylogenetic endemism, meaning the evolutionary history that is unique to an area (Faith et al. 2004), can help improve conservation planning by identifying areas with high or unique levels of evolutionary history (Faith et al. 2004; Faith 1992; Winter et al. 2013).

Another widely used measure is phylogenetic structure (PS), which measures how dispersed or clustered is a community with respect to the tree of life (represented by a larger phylogeny that contains the taxa of interest), than expected by chance (Webb 2000). This is a topology-based measure, which relies on counting mean pairwise nodal distances between taxa in a community (Webb 2000). A suite of null models is used to estimate whether taxa in the community are more or less clustered than expected by chance, providing an idea of the resilience of a given set of taxa. A community or group of taxa with a higher than expected PS, indicating dispersion on the tree of life, has a larger evolutionary diversity and could therefore contain a higher potential to recover from stress or capacity for adaptation (Potter 2008).

In the face of global change, areas of high endemism are particularly sensitive due to the valuable and irreplaceable set of taxa that they host (Margules and Pressey 2000). A given territory is considered an area of endemism when it harbors at least two endemic non-related taxa (Harold and Mooi 1994). At a global scale, endemism of endangered plant taxa is a very important matter to consider. Over 90% of the IUCN, threatened plant species are endemic to a single country (Pittman and Jorgensen 2002) meaning that their preservation depends on a single government conservation policy. The flora of Chile is remarkable in that it harbors the highest percentage of endemicity in South America: four endemic families and 83 endemic genera (Moreira-Muñoz 2011), 67 genera in continental Chile and 16 in the islands. Argentina, for example, has one endemic family and four endemic genera (Zuloaga et al. 1999); Perú, with a flora of over 17,000 vascular plant species, more than three times the flora of Chile, has 51 endemic genera and no endemic family (Brako and Zarucchi 1993). In Ecuador, there are approximately 2110 genera, and only 23 of them are endemic (Jorgensen and León-Yáñez 1999). In an area of similar biogeographic composition such as New Zealand, there are 48 endemic genera (reviewed by Moreira-Muñoz 2011).

Highest levels of floral endemism concentrate in central Chile (Moreira-Muñoz 2011). This is not surprising, given the long-known importance of this area in terms of biodiversity, largely coincident with the location of the “Chilean winter rainfall–Valdivian forest” biodiversity hotspot, with priority for conservation (Armesto et al. 1998; Myers et al. 2000; Sechrest et al. 2002; Arroyo et al. 2004). For example, the families Aextoxicaceae, Gomortegaceae, and Lactoridaceae are restricted to this hotspot (Arroyo et al. 2008). Chile is a very centralized country, with 80% of the population concentrating in central Chile, as well as the main centers for agricultural, industrial, and services activities (INE 2002; CAPP 2008). Despite this, a very small percentage of its area, 5.5% in the north an only 1.7% in the central-southern area, is protected by the National System of Protected Wild Areas (CONAMA 2008). Regarding islands, the flora of the Juan Fernández archipelago, for example, is one of the most vulnerable in Chile and worldwide, mainly due to introduction of alien invading species from the continent, resulting in more than 75% being highly threatened (Swenson et al. 1997). Given the importance of the endemic component and vulnerability of Chilean flora, measures of its evolutionary value become instrumental for aiding conservation efforts.

The explosive growth of bioinformatics and genomics technology provides an unprecedented availability of information that can be used in evolutionary conservation (Roquet et al. 2013). The main objective of this study was to quantify the evolutionary potential of Chilean endemic genera of vascular plants, and its geographic patterns, using as a backbone a previously published vascular plant supertree (Thuiller et al. 2011), and newly added Chilean genera. In addition, the study focused on the relationship between PD and richness at different geographic levels.

## Material and Methods

### Taxon sampling

#### Chilean genera

The complete list of continental and island endemic genera of Chile was obtained from Moreira-Muñoz (2011). It comprises 83 genera, among them 16 pertaining to the Asteraceae, six to the Cactaceae, and four to the Alliaceae. 67 genera occur in the continent and 18 in the Pacific archipelagos Juan Fernández and Islas Desventuradas. Genera *Ochagavia* (Bromeliaceae) and *Notanthera* (Loranthaceae) are both endemic to the islands and to the continent. From these 83 genera, 53 contained DNA sequences in GenBank from where sequences for one representative of each of these 53 genera were obtained. Appendix 1 shows a list of the genera and species used, and their GenBank accession numbers.

A presence/absence matrix for the 53 endemic genera was developed for a geographic one-degree grid, based on data extracted from the National Herbarium of Chile (SGO), complemented in some cases with material from the Herbarium of the University of Concepción (CONC) and own field collections. The database is composed of 2626 records ranging from 20.25 degrees south at Tarapacá coast to 46.81 degrees south in the Aysén region, including offshore islands pertaining to Juan Fernández and Desventuradas archipelagos.

#### Backbone phylogeny

The available genus-level supertree of Thuiller et al. (2011) was used as the backbone phylogeny. This tree was done for European genera, however, from the 378 taxa, 165 are also present in South America, which makes this a representative sample of world vascular flora. Figure S1 shows the backbone phylogeny including the 53 endemic genera of Chile.

### Phylogenetic analyses

Thuiller et al. (2011) used a suite of chloroplast and nuclear markers, assembled into a large matrix, in order to obtain their phylogeny. Alignments for this phylogeny were kindly provided to us by the authors. Matrices were then assembled for the following DNA regions: rbcL, matK, ITS, trnL-trnF, and ndhF. Consequently, the same regions were obtained from GenBank for the Chilean endemic genera, adding these sequences to the large data matrix. As is common in these types of data-mining approaches, not all genes were available for all taxa. However, it has been shown that phylogenetic reconstruction is robust to incomplete data matrices (Driskell et al. 2004).

Alignments were redone to include Chilean genera, using ClustalW (Thompson et al. 1994) and checked to eliminate high homoplasic regions using the software TrimAl v1.2 (Capella-Gutiérrez et al. 2009). Complete data matrices used in this study are available in TreeBase (http://www.treebase.org/treebase-web) and from the authors by request.

Phylogenetic analyses were carried out using maximum likelihood as implemented in the software Garli v0.951 (http://www.bio.utexas.edu/faculty/antisense/garli/Garli.html; Zwickl 2006), especially suited for the analyses of large data matrices. The GTR+I+G model of molecular evolution was used for the combined dataset. One single tree was chosen with the highest –Ln value to perform all the analyses.

### Phylogenetic diversity and phylogenetic structure calculations

The R (R Development Core Team 2012) package Picante (Kembel et al. 2010) was used to calculate phylogenetic diversity and structure. We used phylogenetic diversity (PD) as described by Faith (1992), meaning the sum of branch lengths of the subtree formed by the sampled taxa.

Phylogenetic structure was inferred form a suite of null models, as described by Webb (2000). Mean pairwise distance (MPD) measures the average phylogenetic distance among all pairs of species in a community, reflecting the structure of the whole tree. Mean nearest taxon distance (MNTD) is the average distance between each species in the community and its nearest taxon. This is a measure of the structure close to the tips of the phylogeny (Webb 2000). In order to obtain comparable values among communities, the software Picante calculates standard values for these community structure measures, for a null distribution of communities, generating the standard measures Net Relatedness Index (NRI) for MPD and Nearest Taxon Index (NTI) for MNTD. This is carried out by subtracting the observed values of MPD or MNTD from the average of the null distribution and then dividing by the standard deviation of the null distribution. Positive values of NRI or NTI and high *P*-values (<0.95) mean taxa in the community are more dispersed on the tree of life than expected by chance. Conversely, negative values and low *P*-values (<0.05) mean taxa are more related or clustered than expected by chance.

Phylogenetic diversity and structure measures were obtained for three nested datasets: (1) the whole community of Chilean genera; (2) five regions considered as all grid cells contained in five-latitude degrees from north to south; and (3) each of the 58 grid cells of one degree in which the area of the country into which endemic genera extend was divided.

The 58-cell grid was used to calculated percentages of richness and PD, which were mapped using the software ArcGIS 10 (ESRI 2012).

In addition, phylogenetic endemism (PE; Faith et al. 2004) was calculated as the PD contributed by genera that are strict endemics for each one of the five regions that compose dataset (3), using the same calculation strategy in the software Picante (Kembel et al. 2010) explained for the PD calculation.

### Null models and phylogenetic diversity versus species richness

As the PD metric is mathematically linked to taxon richness, we used the following approaches in order to accurately compare geographic patterns of PD and the relationship between PD and genus richness: (1) our own R script was used to run PD null models consisting of 1000 random sets of genera chosen randomly from the data set of Chilean genera for each grid cell. Random samples were made without replacement to avoid recounting deeper branches. Each random set consisted of the same number of genera that each grid contained originally. For each grid cell, calculated or “real” PD values were compared with the null distribution by subtracting them to the average PD of the null model and dividing by the standard deviation. Positive values indicate grids where calculated PD is larger than expected by chance, and negative values indicate those grids where calculated PD is lower than expected by chance. Statistical significance was obtained with *P*-values. No Bonferroni correction was made as 1000 random samples generate a robust enough distribution to avoid false positives, making it safe to say that results are not stochastic. (2) Discrepancy values were calculated following Pio et al. (2011): PD and richness values for each grid were normalized separately by subtracting the calculated values from the mean of the values obtained for all grids and dividing them by the standard deviation. Normalized PD was then subtracted from normalized richness for each cell. Positive values indicate that for that cell, PD is larger than richness and vice versa for negative values. (3) For the five-latitude degree areas, a linear regression was carried out, in which the residuals were identified geographically, following Forest et al. (2007). Residuals falling above the regression line suggest areas where PD is higher than richness, and those falling below the line suggest areas where PD is lower than richness.

## Results and Discussion

### Phylogeny

Of the 83 endemic genera of Chile, 53 (63%) were represented with one or more DNA sequences in GenBank and were used in the analyses. One best tree with branch lengths was obtained containing 432 taxa. Taxonomic position of genera within the main groups was corroborated using the latest APG classification (http://www.mobot.org/MOBOT/research/APweb/).

### Phylogenetic diversity and structure

#### Chilean flora as a single community

Tables 1 and 2 show the results obtained for PD and null models, and the two measures of community structure, respectively. PD value of Chilean flora as a percentage of total PD doubles the percentage of its richness (meaning the number of Chilean genera with respect to the total number of genera in the tree). PD percentage contributed by the Chilean genera is equivalent to the total phylogenetic endemism (Faith et al. 2004), meaning the amount of PD added to the backbone phylogeny when including all Chilean taxa. Null models indicate that PD obtained for the Chilean taxa is slightly higher than expected by chance (SD 0.1796), but yet not significant (*P*-value 0.999), showing a discrepancy value of 1.34 (Table 1). Measures of community structure, however, indicate no evidence of dispersion. Moreover, taxa seem to be more clustered than dispersed, especially when considering deep branches.

**Table 1:** Results of phylogenetic diversity (PD) measures and species richness (SR) using Chilean endemic genera as a single community.

Community	PD	Mean PD null model (1000 runs)	SD null model	*P*-value	Discrepancy value	% PD^1^	% SR^1^
Chile	10.3213	10.079	0.1796	0.9998	1.3488	24.59	12.27

1 Percentage numbers refer to the PD or generic number of Chilean endemic genera over the total PD or number of genera of the phylogeny, respectively.

**Table 2:** Results of community structure measures using Chilean endemic genera as a single community.

Community	Net Relatedness Index (NRI) (mpd)	*P*-value mpd	Nearest Taxon Index (NTI) (mntd)	*P*-value mntd
Chile	−2.751	0.003	−1.655	0.045
Rest of the tree	1.690	0.96	−3.390	0.0009

#### Geographic patterns of PD and richness

**Figure 1:**
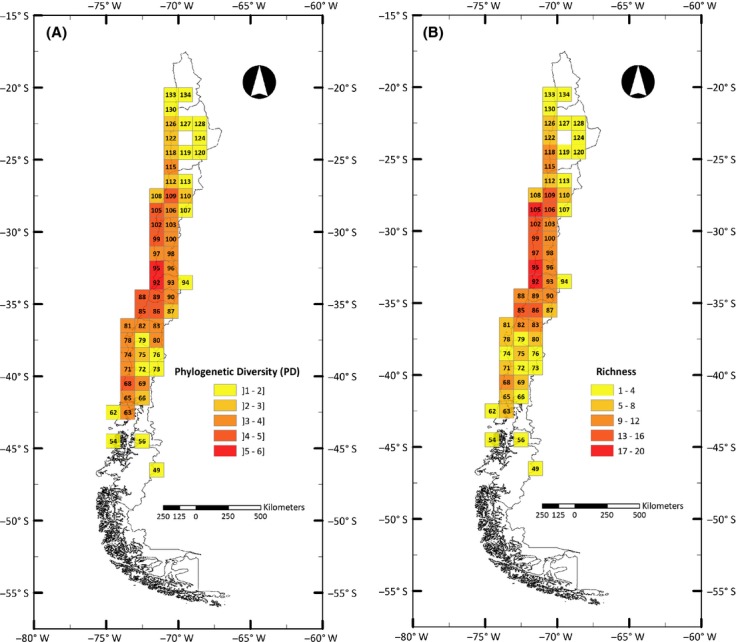
Geographic pattern of calculated phylogenetic diversity (PD) (A) and generic richness (B) mapped onto a one-degree grid along Chile.

**Figure 2:**
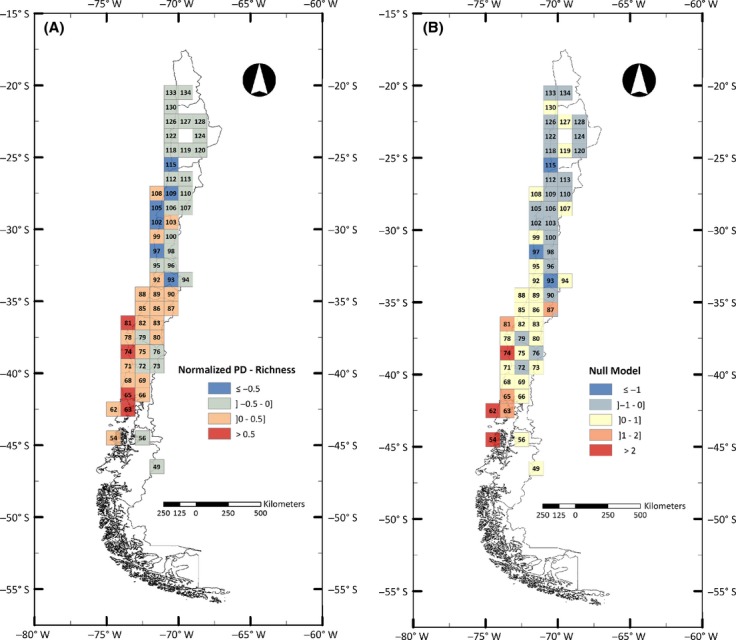
Geographic pattern of phylogenetic diversity (PD) distribution mapped onto a one-degree grid along Chile. (A) Discrepancy values obtained by subtracting normalized generic richness from normalized PD. Positive values represented in shades of red indicate areas where PD is greater than richness, and negative values represented in shades of blue indicate areas where PD is lower than richness. (B) Geographic pattern of a null model approach in which calculated PD for each grid was subtracted to the mean of a 1000 replicates null distribution and divided by the standard deviation of the distribution. Positive values indicating that PD is larger than expected by chance are represented in shades of red; negative values indicating that PD is lower than expected by chance are represented in shades of blue.

The general distribution pattern of generic richness in one-degree grid cells coincides largely with the pattern observed for PD, both concentrating in central Chile and decreasing to the north and south (Fig. 1). As the PD value is mathematically dependent on taxon richness, we normalized PD and richness values in order to eliminate richness biases from PD patterns. This analysis showed an interesting geographic pattern (Fig. 2A) in which areas where the normalized value tends to be positive, indicating that PD is larger than richness, concentrate in southwestern areas of Chile, whereas northern areas tend to show the opposite pattern, meaning that in general, normalized PD for those areas appears to be lower than normalized richness.

An interesting strip where normalized PD is higher than normalized richness can be observed for the coastal area between Talca and the Chiloé island (34–42 degrees south), coinciding with part of the coastal Valdivian temperate rainforest. This is a complex open forest with many species that are unique to the region (Tecklin et al. 2011). Coastal areas in Chile are leftacterized by hosting populations that are in general older than in central Chile or the Andes that have been regarded as isolated systems due to the influence of the arid diagonal that crosses South America SE–NW, which has resulted in marked levels of endemisms and unique diversity (Villagran and Hinojosa 1997; Villagrán and Armesto 2005).

During glaciation periods, many species restricted their range to a few refuges mostly located in southern areas, which constitute valuable areas for the long-term survival of lineages, which expanded after the ice melted (Taberlet and Cheddadi 2002). In the Los Lagos Region (40–44 degrees south), including the Chiloé island, evidence shows that the ice covered the Andes and Central valley. The coastal mountain acted in this area as a refuge for many plant taxa (Armesto et al. 1994; Villagrán et al. 1995; Villagrán 2001). Sérsic et al. (2011), for example, found phylogeographic patterns in plants and vertebrates that suggest stable areas where species would have survived the last glacial maximum, located in the coastal areas between 36° and 41° south and in the north of the Chiloé island.

Modern floras in Chile have been related to paleofloras that are generally older as latitude increases. In this way, Hinojosa (2005) describes for the south of Chile an Eocene to early Miocene (55–20 Mya) mixed flora with elements of the Austral-Antarctic floristic element. The Coastal range floras (36–37 degrees south) resemble this mixed paleofloras, dated some 23 Mya. To the north, Neogene subtropical paleoflora is likely the ancestor of central Chile forests 32–33 degrees south: ancestors of extant sclerophyllous forests in central Chile have been dated some 20–15 Mya (Hinojosa et al. 2006). In central-northern Chile, by the mid-Miocene (15 Mya), the Andes mountain reached about half its altitude, possibly already exerting a rain-shadow effect (Gregory-Wodzicki 2000; Schlunegger et al. 2010). Semi-arid to arid climate, based on sedimentological evidence, prevailed in the central Andes from 15–4 Mya; in the northernmost area of Chile, hyperaridity is attributed to this pre-existing arid condition and the onset of the Humboldt current 3.5–3 Mya (Hartley 2003).

Our null model approach, in which we analyzed whether the value of PD obtained per grid cell was higher or lower than expected by chance, showed a very similar geographic pattern to the one observed for the analysis of normalized PD versus normalized richness. This means that in absolute values, the southwestern area showed more grids in which PD was higher than expected by chance (Fig. 2B), and the opposite was seen for the northern area. This agrees with studies mentioned previously in which the floras of the southern area are generally older than the more xeric ones in Chile. One would expect then that older areas that also acted as glacial refuges would harbor taxa that accumulate more evolutionary history, which would explain the results of the null model approach and also the discrepancy between PD and richness. However, the null model results have to be looked at with caution, as very few of the values were statistically supported. Interestingly, significant *P*-values (*P* < 0.05) were only observed for three grids located in the south area of Chile, encompassing a strip of the coastal area and part of the Chiloé island (grids 54, 62 and 74), coinciding with the areas described as glacial refuges previously. The northern grids, even though showing negative values, were not statistically significant for the null model, meaning that their calculated PD was not statistically lower than expected by chance.

**Figure 3:**
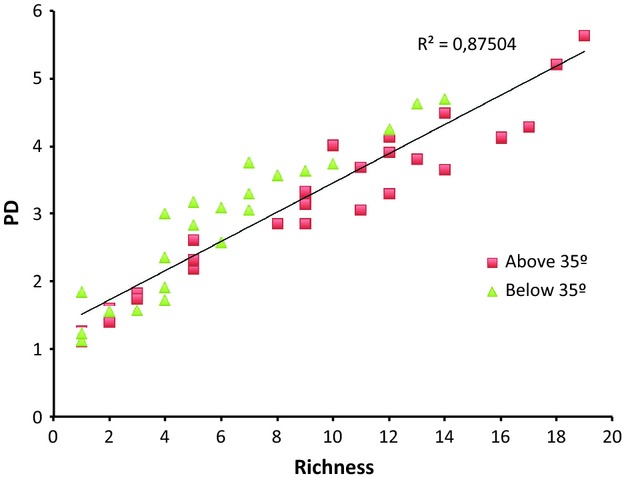
Linear regression between phylogenetic diversity (PD) and generic richness for five latitudinal intervals of five degrees each. Residuals of the regression were clustered into two groups: one encompassing all intervals from 35 degrees and above (north), represented as red squares and the second one encompassing all intervals from 35 degrees and below (south) represented as green triangles.

An additional geographic scale was explored by dividing the country into five latitudinal intervals of five degrees each (last one considers ten degrees as only one grid falls outside of the 5-degree interval). The relationship between PD and richness is expressed as a linear regression, in which the residuals are color-coded by geographic area (Fig. 3). The results show that in fact, the residuals of the northern areas (above 35 degrees south) mostly fall below the regression line, whereas areas to the south (below 35 degrees south) fall mostly above it. An intermediate situation occurs in central Chile (30–35 degrees), where residuals locate in both sides of the regression (result not shown). Phylogenetic structure measures coincide with this (Table 3), suggesting statistically significant clustering of the endemic genera in the 25–30 degree interval both close to the tips of the tree as well as for deep branches. For the 20–25 degree interval, clustering is observed closer to the tips of the tree, whereas the 30–35 degree interval shows phylogenetic clustering for deep branches. Southern areas do not show statistically significant phylogenetic clustering at any level, nor do they show any evidence of over-dispersion.

**Table 3:** Community structure measures of Chilean endemic vascular plant genera in five-degree latitude intervals.

Latitude degrees interval	Community structure			
	Net Relatedness Index (NRI) (mpd)	*P*-value mpd	Nearest Taxon Index (NTI) (mntd)	*P*-value mntd
20–25	−1.167	0.1578	−16.55^*^	0.036
25–30	−2.368^*^	0.0019	−24.06^*^	0.004
30–35	−1.844^*^	0.033	−0.82	0.206
35–40	−1.151	0.126	−0.83	0.196
40–50	−1.094	0.166	−14.37	0.080

Asterisks shows statistically significant values (*P* < 0.05).

**Figure 4:**
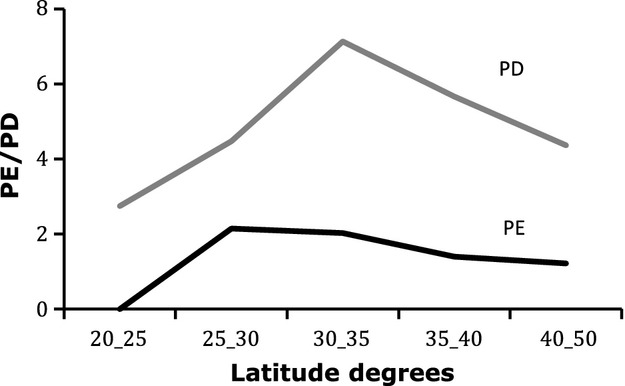
Phylogenetic diversity (PD) and phylogenetic endemism (PE) versus latitude as calculated for five areas of five degrees each along Chile. The upper line represents PD per area, calculated as the sum of branch lengths of all the endemic Chilean genera inhabiting the area. The lower line represents PE, calculated as the sum of branch lengths of the genera that exclusively inhabit in each of the five areas.

Phylogenetic endemism was calculated for the four five-degree macro areas, as the sum of the branch lengths encompassed by the genera that are strict endemics of each of the five areas (Faith et al. 2004). Results show a pattern that differs from that of PD for each of the areas (Table 4), evidencing a shift in the peak of endemism toward the north of the country, between 25 and 30 degrees south (Fig. 4). Previous studies of floral richness using latitudinal gradients at different taxonomic levels also find differences between peaks of richness and peaks of endemism. Family, genus, and species richness concentrates in central Chile and decreases to the north and south (Bannister et al. 2012), as is also observed for PD in this study. The same authors find a peak of endemism at all taxonomic levels between 22 and 37 degrees south, partly coincident with the peak in PE found in this study.

**Table 4:** Phylogenetic diversity (PD) and phylogenetic endemism (PE) calculated for each of the five latitudinal areas defined as strips of five degrees.

Latitude degrees interval	PD	Number of taxa per region	PE	Number of strict endemic taxa per region
20–25	2.75	8	0.00	0
25–30	4.46	21	2.14	5
30–35	7.13	31	2.02	4
35–40	5.67	20	1.40	2
40–50	4.35	13	1.22	1

Evolutionary measures such as phylogenetic diversity and community structure can aid the evaluation of taxa and ecosystems for conservation purposes. Our study has shown interesting geographic patterns of the spatial structure of PD values in a highly endemic territory. Even though the addition of more taxa will be useful for a more complete overview, we believe that there are areas of Chile that harbor a larger evolutionary history than others and therefore should be looked at more closely, especially when designing conservation strategies.

## APPENDIX

**Appendix 1:** Chilean endemic genera and species used in this study, with their GenBank accession numbers.

Genus	Species	rbcL	trnL-F	matK	ITS	ndhF
*Adenopeltis*	*serrata*	AY794844	AY794633	AB268058		
*Balsamocarpon*	*brevifolium*	AB268058	AF430761	EU361864	AY308548	
*Bridgesia*	*incisifolia*	AY724347	EU721435	EU720645	EU720476	
*Calopappus*	*acerosus*				FJ979685	
*Centaurodendron*	*palmiforme*		JF754757		JF754806	
*Conanthera*	*bifolia*	JX903230				
*Copiapoa*	*bridgesii*			AY015293		DQ855879
*Cyphocarpus*	*rigescens*	L18792				
*Dendroseris*	*litoralis*			DQ840434	AJ633305	
*Desmaria*	*mutabilis*	EF464527	DQ340603	EF464509	DQ333852	
*Dinemagonum*	*gayanum*	AF344468		HQ247265		AF351084
*Dinemandra*	*ericoides*	AF344469	AF351002	AF344542		AF351069
*Ercilla*	*volubilis*	AJ235800		AY042583		
*Eriosyce*	*islayensis*		JF975709	AY015337	AY064351	
*Eulychnia*	*acida*		AY566663			
*Fascicularia*	*bicolor*		FJ942957	AY950023		HQ895749
*Francoa*	*appendiculata*		DQ452889			
*Gomortega*	*nitida*		AF012404		AF289846	
*Guynesomia*	*scoparia*				DQ479035	
*Gymnachne*	*koelerioides*		EU395896	DQ786918	EU395919	DQ786846
*Gypothamnium*	*pinifolium*	EU736105	EU729338		EU729342	EU729346
*Homalocarpus*	*dissectus*	DQ133812		DQ133791		
*Huidobria*	*fruticosa*		AY254247	AY254064		
*Ivania*	*cremnophila*				HQ541176	
*Juania*	*australis*	AJ829874	AM113638	AM114608		
*Jubaea*	*chilensis*	AJ829875	EU004863	EU004869		EU004878
*Lactoris*	*fernandeziana*	L08763	AY145342	DQ882195		
*Lapageria*	*rosea*	Z77301		AY624480		AY225008
*Latua*	*pubiflora*		EU581011			EU580903
*Legrandia*	*concinna*			AM489990	AM234072	
*Leonotochir*	*ovallei*	AY120369				AY224996
*Leucocoryne*	*coquimbensis*	Z69199	AF508518			AF508407
*Megalachne*	*berteroniana*				FR692028	
*Miquelopuntia*	*miquelii*		HM041303	HM041722		
*Mosleftia*	*solbrigii*		EF530306		EF530219	
*Notanthera*	*heterophylla*	EF464529	DQ340606	EF464511	DQ333855	
*Ochagavia*	*carnea*	JF280726	HQ882744	EU681905		HQ895755
*Oxyphyllum*	*ulicinum*	EU736104	EU729340		EU729344	EU729348
*Peumus*	*boldus*	AF206807	AF012403	AJ247183	GU177689	
*Phrodus*	*microphyllus*	FJ914176	FJ189725		FJ439765	EU742306
*Pintoa*	*chilensis*	AJ133858	AJ387954			
*Placea*	*arzae*				FJ349191	FJ264228
*Podophorus*	*bromoides*				FR692035	
*Robinsonia*	*gracilis*		EF538118	GU817514	EF538290	
*Sarmienta*	*repens*		AY364264		EF445701	U62194.2
*Scyphanthus*	*elegans*		AY254298	AF503334		
*Tecophilaea*	*cyanocrocus*	HM640544	AJ290310	HM640661		
*Thyrsopteris*	*elegans*			JF303910		
*Traubia*	*modesta*	AF116986	AF104748			FJ264237
*Trevoa*	*quinquinervia*		AY642155			
*Valdivia*	*gayana*	AJ575924	GQ984052		GQ983642	AJ419703
*Vestia*	*lycioides*		AY206769	EF438822		AY206751
*Zephyra*	*elegans*	Y17340	AJ290311	AJ579994		

## Supporting Information

Figure S1 Backbone phylogeny used (Thuiller et al. 2011). Chilean endemic genera are highlighted in red.
