# Supplementary material for: Endemicity and evolutionary value: a study of Chilean endemic vascular plant genera
Source: Ecol Evol. 2014 Feb 19;4(6):806–16. doi: 10.1002/ece3.960 (PMC3967905; doi:10.1002/ece3.960)
Supplement: Figure S1 — Backbone phylogeny used (Thuiller et al. 2011). Chilean endemic genera are highlighted in red. [file ece30004-0806-sd1.docx]

Figure S1. Backbone phylogeny used (Thuillier et al 2011). Chilean endemic genera are highlighted in red.
